# Association Between Serum Lipid Profile and Obstructive Respiratory Events During REM and Non-REM Sleep

**DOI:** 10.1007/s00408-019-00195-7

**Published:** 2019-02-02

**Authors:** Andras Bikov, Zsofia Lazar, Peter Horvath, David Laszlo Tarnoki, Adam Domonkos Tarnoki, Luca Fesus, Marton Horvath, Martina Meszaros, Gyorgy Losonczy, Laszlo Kunos

**Affiliations:** 1 Department of Pulmonology, Semmelweis University, 1/C Dios arok, Budapest, 1125 Hungary; 2 Department of Radiology, Semmelweis University, 78/A Ulloi ut, Budapest, 1082 Hungary

**Keywords:** Apolipoproteins, Dyslipidaemia, Lipids, Obstructive sleep apnoea, REM sleep

## Abstract

**Purpose:**

Obstructive sleep apnoea (OSA) represents a risk for dyslipidaemia. Obstructive respiratory events during rapid eye movement (REM) sleep are more strongly related to the development of hypertension and diabetes than in non-REM. However, the relationship between sleep phases and serum lipid profile is unclear. We aimed to analyse the relationship between obstructive respiratory events in REM and non-REM sleep as well as serum lipid profile.

**Methods:**

Polysomnography was performed in 94 adult subjects who did not take any lipid-modifying medications. Fasting venous blood sample was taken the following morning for total cholesterol, triglyceride, high-density lipoprotein cholesterol (HDL-C), low-density lipoprotein cholesterol, lipoprotein(a), apoprotein A1 (ApoA1) and for apoprotein B (ApoB) measurements. Lipid profiles were correlated with apnoea–hypopnoea index (AHI) during REM (AHI_REM_) and non-REM (AHI_NREM_) stages in all subjects. In addition, lipid profiles were compared between REM-dependent OSA patients (AHI_REM_ ≥ 5/h, but AHI_NREM_ < 5/h) and control subjects (both AHI_REM_ and AHI_NREM_ < 5/h).

**Results:**

AHI_REM_ correlated only with triglyceride concentrations (*p* = 0.04, Spearman’s rho, *ρ* = 0.21). In contrast, there was a significant association between AHI_NREM_ and triglyceride (*p* = 0.02, *ρ* = 0.23), ApoB (*p* = 0.03, *ρ* = 0.21), HDL-C (*p* < 0.01, *ρ* = − 0.32) as well as ApoA1 levels (*p* = 0.04, *ρ* = − 0.21). However, these correlations were not present after adjustment for BMI (all *p* > 0.05). There was no difference in the lipid profile of REM-dependent OSA subjects and healthy controls (*p* > 0.05).

**Conclusions:**

Altered serum lipid profile is equally associated with a disturbed REM and non-REM sleep in OSA. Obesity must be considered as a strong covariate when interpreting lipid data in sleep apnoea.

## Introduction

Obstructive sleep apnoea (OSA) is a common disorder which is characterised by the repetitive episodes of total or partial collapse of pharynx leading to intermittent hypoxia and frequent micro-arousals. On one hand, patients with OSA commonly report daytime sleepiness and impaired performance in daily activities. On the other hand, this disorder is associated with the development and worsening of cardiovascular and cognitive diseases as well as metabolic dysfunction [1] such as dyslipidaemia [2, 3].

Dyslipidaemia is a major risk factor in the development of cardiovascular diseases [4]. Although traditionally, total cholesterol, low-density lipoprotein cholesterol (LDL-C), high-density lipoprotein cholesterol (HDL-C) and triglyceride (TG) are the main lipid components measured in clinical practice, guidelines recommend the analysis of additional components including apoprotein A1 (ApoA1), apoprotein B (ApoB) and lipoprotein(a) (LPA) when assessing dyslipidaemia [4]. Dyslipidaemia is highly prevalent in OSA [5] and large cohort studies showed elevated total cholesterol and TG as well as lowered HDL-C levels [6, 7].

Chronic intermittent hypoxia and fragmented sleep architecture contribute to dyslipidaemia independently [1]. Chronic intermittent hypoxia blocks lipoprotein lipase via hypoxia-inducible factor leading to increased levels of TG [1]. Furthermore, inflammation and oxidative stress in OSA result in altered structure of visceral white adipose tissue [8]. Of note, sleep fragmentation increases adipocyte number and size [1]. OSA also shifts appetite towards increased consumption of fat and carbohydrate by reducing the concentration of leptin and elevating the level of ghrelin in plasma [1, 9]. An increase in sympathetic activity, a consequence of hypoxia and micro-arousals may liberate cholesterol and TG from adipocytes [10]. Moreover, oxidative stress burden in OSA accelerates the development of cardiovascular disease related to lipid dysfunction [3]. Finally, OSA induces alterations in the production of thyroid [11] and growth hormones [12], which influence lipid metabolism.

Recent evidence suggests that the association between OSA and the development of comorbidities, such as cardiovascular disease [13], hypertension [14–16], atherosclerosis [17, 18] and diabetes [19], strongly depends on the number of apnoeic events and desaturations during rapid eye movement (REM) sleep. It is known that the production of growth hormone (GH), cortisol or thyroid hormones during night shows variability between REM or non-REM sleep [11, 20]. In addition, compared to non-REM sleep, REM sleep is characterised by higher sympathetic tone [21] in all subjects and longer apnoeic periods as well as greater oxygen desaturations in patients with OSA [22]. As hypoxia and augmented sympathetic activity may exaggerate dyslipidaemia [3], one would expect that REM sleep-related obstructive respiratory events induce more prominent changes in the lipid profile compared to the non-REM sleep. However, this has not been investigated before.

Therefore, the aim of our study was to analyse the relationship between obstructive respiratory events during REM and non-REM sleep on the lipid profile.

## Methods

### Study Design and Subjects

Ninety-four adult volunteers were recruited for the study [49 ± 16 years, 30 men, body mass index (BMI) of 26.4 ± 5.2 kg/m^2^]. 13 subjects (14%) were current or ex-smokers, 41 (44%) volunteers had hypertension and 12 (13%) were treated with diabetes. Subjects taking any anti-lipid treatment, including statins, fibrates or nicotinic acid, being on ketogenic diet and those with known primary dyslipidaemia were excluded. None of the patients had been diagnosed with OSA and had not used continuous positive airway pressure (CPAP) or oral appliance device.

After taking medical history and filling out the Epworth sleepiness scale (ESS), subjects attended a full-night polysomnography. The following morning, fasting venous blood was collected for C-reactive protein (CRP), total cholesterol, TG, HDL-C, LDL-C, LPA, ApoA1 as well as ApoB measurements before taking any medications.

The study was approved by the local Ethics Committee (Semmelweis University, TUKEB 30/2014) and informed consent was obtained from all participating volunteers. The study was conducted according to the Declaration of Helsinki (as revised in Brazil 2013).

### Polysomnography

Polysomnography was performed as described previously [23] using SOMNOscreen Plus Tele PSG (SOMNOmedics GMBH Germany). Briefly, electroencephalogram, electrooculogram and electromyogram, thoracic and abdominal respiratory excursions, breath sounds, nasal pressure, electrocardiogram and oxygen saturation were registered [24]. Sleep stages, movements and cardiopulmonary events were scored manually according to the American Academy of Sleep Medicine (AASM) guidelines [25]. Total sleep time (TST), sleep period time (SPT), percentage of total sleep time spent in rapid eye movement stage (REM%), percentage of total sleep time spent with saturation below 90% (TST90%) and minimal O_2_ saturation (minSatO_2_) were recorded; Apnoea-hypopnoea index (AHI), oxygen desaturation index (ODI) and arousal index (AI) were calculated. AHI was evaluated both during REM (AHI_REM_) and non-REM (AHI_NREM_) sleep. Obstructive sleep apnoea was defined as having an AHI ≥ 5/h, while REM-dependent OSA as having an AHI_REM_ ≥ 5/h, but AHI_NREM_ < 5/h.

### Statistical Analysis

Statistica 12 (StatSoft, Inc., Tulsa, OK) software was used for statistical analysis. Data normality was tested with the Kolmogorov–Smirnov’s test. Categorical parameters were compared with Chi-square test. Lipid profile was compared between the OSA and non-OSA groups (AHI < 5/h) as well REM-dependent OSA and controls (both AHI_REM_ and AHI_NREM_ < 5/h) using the Mann–Whitney test. The Spearman’s test was used to correlate lipids with clinical parameters. The effects of BMI, age and gender as co-variates were assessed with multivariate analyses and the general mixed linear model. A *p* value < 0.05 was considered statistically significant.

## Results

### Patient Characteristics

Following polysomnography, the volunteers were divided into OSA (*n* = 41) and non-OSA (*n* = 53) groups. Patients with OSA were classified into mild (AHI 5–14.9 events/h, *n* = 21), moderate (AHI 15–29.9 events/h, *n* = 13) and severe (AHI > 30 events/h, *n* = 7) subgroups. The patients were significantly older, had higher BMI, had higher prevalence of males and hypertension (all *p* < 0.05). They also had longer total sleep time and sleep period time, higher AHI, ODI, TST90% and lower MinSatO_2_ (all *p* < 0.05). There was no difference in the prevalence of smokers or patients with diabetes, CRP, ESS, REM% or AI (all *p* > 0.05) between the two groups. Subjects’ characteristics are summarised in Table 1.

**Table 1:** Subject’s characteristics

	Control (*n* = 53)	OSA (*n* = 41)	*p* Value
Gender (male/female)	12/41	18/23	**0.02**
Age (years)	45 (20–74)	60 (29–74)	< **0.01**
BMI (kg/m^2^)	24.6 ± 3.6	28.7 ± 6.1	< **0.01**
Hypertension (%)	21	61	< **0.01**
Diabetes (%)	17	9	0.27
Smokers (%)	20	9	0.16
Epworth sleepiness scale	6 (1–14)	6 (0–14)	0.62
CRP (mg/ml)	1.8 (0.2–73.2)	2.3 (0.2–49.5)	0.58
Total cholesterol (mmol/l)	5.30 (3.50–8.50)	5.40 (3.40–8.50)	0.73
HDL-C (mmol/l)	1.72 (0.79–3.74)	1.48 (0.88–5.97)	< **0.01**
LDL-C (mmol/l)	3.03 (1.09–5.43)	3.11 (1.36–5.57)	0.50
TG (mmol/l)	1.00 (0.42–3.99)	1.34 (0.65–4.16)	< **0.01**
ApoA1 (g/l)	1.62 (0.00–2.10)	1.51 (0.84–2.29)	0.07
ApoB (g/l)	1.06 (0.45–3.22)	1.23 (0.69–2.12)	**0.04**
LPA (g/l)	0.12 (0.00–1.13)	0.31 (0.00–1.13)	0.32
TST (min)	383 ± 57	412 ± 35	< **0.01**
SPT (min)	418 ± 50	447 ± 36	< **0.01**
REM%	15.6 ± 7.0	16.3 ± 6.7	0.64
AHI (1/h)	2.3 (0.0–4.8)	14.6 (5.0–126.5)	< **0.01**
ODI (1/h)	0.9 (0.0–4.3)	11.3 (1.5–120.60)	< **0.01**
TST90%	1.5 ± 6.7	7.9 ± 19.5	< **0.01**
MinSatO_2_	90.7 ± 3.3	83.0 ± 8.4	< **0.01**
AI (1/h)	46.8 ± 20.1	47.0 ± 20.8	0.91

The data are expressed as mean ± standard deviation for parametric and median (range) for non-parametric variables. Significant differences are presented in bold

*BMI* body mass index, *CRP* C-reactive protein, *HDL-C* high-density lipoprotein cholesterol, *LDL-C* low-density lipoprotein cholesterol, *TG* triglyceride, *ApoA1* apolipoprotein A1, *ApoB* apolipoprotein B, *LPA* lipoprotein (a), *TST* total sleep time, *SPT* sleep period time, *REM%* percentage of total sleep time spent in rapid eye movement stage, *AHI* apnoea–hypopnoea index, *ODI* oxygen desaturation index, *TST90%* percentage of total sleep time spent with saturation below 90%, *MinSatO*_*2*_ minimal O_2_ saturation, *AI* arousal index

### The Lipid Profile in the OSA and Control Groups

Serum triglyceride and ApoB levels were higher, while serum HDL-C levels were lower in OSA (all *p* < 0.05). There was a trend for lower ApoA1 concentrations in OSA (*p* = 0.07). There was no difference in total cholesterol, LDL-C or LPA between the two groups (*p* > 0.05, Table 1). Correlation between the elements of lipid profile and clinical variables is shown in Table 2.

**Table 2:** Relationships between serum lipid levels and clinical variables

	Total cholesterol (mmol/l)	HDL-C (mmol/l)	LDL-C (mmol/l)	TG (mmol/)	ApoA1 (g/l)	ApoB (g/l)	LPA (g/l)
Gender (male)	NS	− **0.46**	NS	**0.25**	− **0.32**	NS	NS
Age (years)	**0.28**	NS	NS	**0.23**	NS	**0.23**	NS
BMI (kg/m^2^)	**0.21**	− **0.42**	**0.33**	**0.49**	NS	**0.32**	NS
Hypertension	NS	− **0.26**	NS	**0.42**	NS	NS	NS
Diabetes	NS	NS	NS	**0.27**	NS	**0.31**	NS
Smoker	NS	NS	NS	NS	NS	NS	NS
ESS	NS	NS	NS	**0.26**	NS	NS	NS
CRP (mg/ml)	**0.33**	− **0.27**	**0.36**	**0.35**	NS	**0.46**	NS
TST (min)	NS	NS	NS	**0.31**	NS	NS	NS
SPT (min)	NS	NS	NS	NS	NS	NS	NS
REM%	NS	NS	NS	NS	NS	NS	NS
AHI (1/h)	NS	− **0.29**	NS	**0.26**	NS	**0.21**	NS
ODI (1/h)	NS	− **0.38**	NS	**0.39**	NS	**0.29**	NS
TST90%	**0.25**	− **0.23**	**0.22**	**0.35**	NS	**0.29**	NS
MinSatO_2_	− **0.29**	**0.31**	− **0.33**	− **0.37**	NS	− **0.31**	NS
AI (1/h)	NS	NS	− **0.24**	NS	NS	NS	NS

Spearman’s rho are presented for significant relationships in bold. Non-significant associations are marked as NS

*HDL-C* high-density lipoprotein cholesterol, *LDL-C* low-density lipoprotein cholesterol, *TG* triglyceride, *ApoA1* apolipoprotein A1, *ApoB* apolipoprotein B, *LPA* lipoprotein (a), *BMI* body mass index, *CRP* C-reactive protein, *ESS* Epworth sleepiness scale, *TST* total sleep time, *SPT* sleep period time, *REM%* percentage of total sleep time spent in rapid eye movement stage, *AHI* apnoea–hypopnoea index, *ODI* oxygen desaturation index, *TST90%* percentage of total sleep time spent with saturation below 90%, *MinSatO*_*2*_ minimal O_2_ saturation, *AI* arousal index

Differences in serum triglyceride, ApoB and HDL-C levels between OSA and control groups were adjusted for BMI, age and gender. After adjustment for BMI, the differences between the two groups for the three lipid parameters became insignificant (all *p* > 0.05). Adjustment for male gender significantly affected the difference in HDL-C, while adjustment for age did not influence any of the inter-group differences.

### Relationship Between Lipid Profile and AHI_REM_ as well as AHI_NREM_

**Fig. 1:**
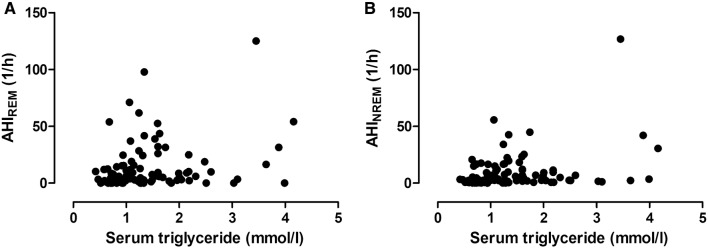
Relationship between serum triglyceride levels and AHI_REM_ (**a**) as well as AHI_NREM_ (**b**). There was a significant correlation between TG levels and AHI_REM_ (*p* = 0.04, *ρ* = 0.21, **a**) and AHI_NREM_ (*p* = 0.02, *ρ* = 0.23, **b**)

**Fig. 2:**
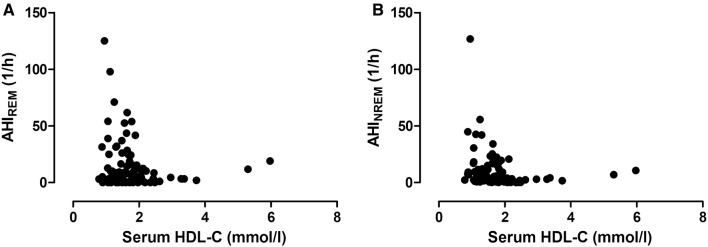
Relationship between serum HDL-C levels and AHI_REM_ (**a**) as well as AHI_NREM_ (**b**). There was no correlation between HDL-C and AHI_REM_ (*p* > 0.05, **a**), while there was a significant relationship between HDL-C and AHI_NREM_ (*p* < 0.01, *ρ* = − 0.32, **b**)

There was a significant correlation between AHI_REM_ and TG levels (*p* = 0.04, *ρ* = 0.21, Fig. 1a), but not with any other lipid components (Fig. 2a). In contrast, AHI_NREM_ correlated directly with TG (*p* = 0.02, *ρ* = 0.23, Fig. 1b) and ApoB (*p* = 0.03, *ρ* = 0.22), and indirectly with HDL-C (*p* < 0.01, *ρ* = − 0.32, Fig. 2b) and ApoA1 (*p* = 0.04, *ρ* = − 0.21).

Adjusting for BMI, all the relationships above became insignificant (*p* > 0.05). After adjustment for male gender the relationships between AHI_NREM_ and ApoA1, ApoB and HDL-C became insignificant, but this did not affect the associations between TG and AHI_REM_ or AHI_NREM_. Adjustment for age did not affect the relationship between AHI_REM_ and TG, AHI_NREM_ and TG as well as AHI_NREM_ and HDL-C.

### Differences Between REM-Dependent OSA and Control Groups

From all volunteers, 16 were diagnosed with REM-dependent OSA. Their lipid profile was compared with control subjects (*n* = 39). There was no difference in any of the investigated lipid parameters (all *p* > 0.05, data not shown).

## Discussion

In this study, we evaluated the relationship between obstructive respiratory events during REM versus non-REM sleep and lipid profile. We found that TG levels related to AHI irrespective of REM or non-REM phase, while HDL-C, ApoA1 and ApoB were associated with AHI only during non-REM stage. In contrast to cardiovascular disease, hypertension and diabetes, the presence of dyslipidaemia in OSA may not be influenced by the timely occurrence of respiratory events.

The need for biomarkers which predict OSA-related mortality has been emphasised [26], and the measurement of serum lipids may have additional clinical value when predicting long-term consequences such as cardiovascular morbidity and mortality. Dyslipidaemia is a major factor in the development of cardiovascular diseases, and OSA is associated with altered lipid profile. According to the European Society of Cardiology/European Atherosclerosis Society, dyslipidaemia should be evaluated based on the profile of multiple lipids and lipoproteins [4]. The measurement of apoA1 and apoB have advantages over conventional analyses of lipids, namely, that the assays do not require fasting conditions and are not influenced by high TG levels [4]. ApoB is the major constituent of the atherogenic lipoproteins, such as LDL, very low-density lipoprotein and intermediate density lipoprotein, while apoA1 is the main component of HDL. Despite the aforementioned methodological advantage, it seems that the measurement of apoA1 and apoB does not provide additive clinical value over traditional lipid levels [4]. LPA has a similar structure to LDL; however, it contains a unique apolipoprotein, the apolipoprotein (a). It may pose further risk for cardiovascular disease in subjects with family history of thromboembolic events [4].

Studies investigating serum lipid profile in OSA are contradictory. In general, reports finding no association analysed data from a limited number of subjects [27]. However, in case–control studies recruiting many volunteers, the relationship between OSA severity and lipid levels was significant, but it was also weak [6, 7] and non-linear [2]. A meta-regression analysis by Nadeem et al. concluded that OSA is associated with high total cholesterol, LDL-C and TG and low HDL-C [28]. Nevertheless, even if a study did not find a difference in lipid or apolipoprotein concentrations, dysfunctionality of HDL was reported, suggesting qualitative rather than quantitative alterations in lipid profile [29]. A study involving more than 500 subjects demonstrated a direct relationship between AHI and apoB levels but no relationship with apoA1 [30]. On contrary, Guan et al. reported a direct relationship with apoB and an indirect association with apoA1 in nearly 3000 volunteers [2]. Considering all previous data, the difference in serum lipid and apoprotein concentrations between the OSA and control groups is small and may be statistically significant only when increasing the number of subjects [28]. We found a significant difference in TG, HDL-C and ApoB, while ApoA1 tended to be lower in OSA. Interestingly, there was a significant association between markers of hypoxaemia and LDL-C as well as total cholesterol, while these lipids did not relate to AHI or ODI. These confirm the previous data that hypoxia is more strongly related to dyslipidaemia than to the apnoeic periods [7, 30]. In line with previous reports [2], LPA levels were unaltered in OSA in the current study. Confirming the recent data on the association between systemic inflammation and dyslipidaemia in OSA [31], lipid markers except for ApoA1 and LPA correlated with CRP.

Although animal models show that chronic intermittent hypoxia and sleep fragmentation may independently induce lipid dysfunction [1], this has not been confirmed by the present study. Only LDL-C correlated with arousal index, suggesting that a more fragmented sleep is associated with lower LDL-C. These surprising associations need to be confirmed in an independent cohort.

The significance of obesity as a covariate is frequently disputed [1]. In experimental OSA models, dyslipidaemia was induced in non-obese animals [1], and in some human studies the relationship between OSA severity and dyslipidaemia existed even after adjustment for BMI [2, 7, 30]. A recent study involving more than 8000 participants reported that paradoxically, morbid obesity may be associated with lower TG and higher HDL-C [7]. As OSA severity is directly related to BMI [32], obesity is likely to be a bias for the relationship between OSA and lipids. In the current work, similarly to a recent study conducted on 2983 patients [2], obesity was found to be a significant covariate; however, the extent of its effect needs to be estimated in larger cohorts. Of note, the relationship between obesity and OSA is bidirectional. On one hand, obesity worsens OSA by inducing accumulation of fat tissue around upper airways and influencing ventilatory control [33]. On the other hand, sleep fragmentation promotes hyperphagia and increases the number and size of adipocytes, while chronic intermittent hypoxia induces lipolysis and inflammation in fat tissue [1]. Nevertheless, after the adjustment for BMI, all correlations between OSA severity and lipid markers became insignificant. Waist circumference may a better estimate for obesity than BMI [34]. However, we have not measured that in this study.

The lipid profile shows geographic, racial, gender- and age-related variability [34]. In addition, OSA is more strongly related to metabolic syndrome in younger age [35]. However, adjustment for age influenced our results only marginally, while only the relationships between AHI and TG remained significant after adjustment for gender.

Because of the prominent effect of BMI, the conclusions on AHI_REM_ as well as AHI_NREM_ need to be interpreted carefully. Previous studies finding AHI_REM_ dependence of hypertension and insulin resistance concluded that the prominent effect of REM sleep may be due to increased sympathetic tone [14–16, 19]. In fact, increased sympathetic activity may induce lipolysis liberating circulating lipids [3]. However, it seems that this effect is marginal in OSA as non-REM-related sleep disturbances were more closely related to dyslipidaemia. This is in line with the previous results showing that although CPAP reduces sympathetic activity, it has only a minor effect on the metabolic profile [36]. Apart from the sympathetic activity, significant sleep phase-related differences exist also in the production of hormones which may affect lipid metabolism. It is known that GH is mainly produced during stage N3 sleep [20] and OSA is associated with decreased levels of GH [12]. Exogenous GH has been reported to increase HDL-C in growth hormone-deficient patients [37] suggesting a direct relationship between GH and HDL-C. Additionally to intermittent hypoxia and sleep fragmentation, the increased production of free fatty acids in OSA may itself decrease GH production [12]. The release of another hormone, the thyrotropin also peaks during early, non-REM dominant stages of sleep with a significant reduction during sleep deprivation [11]. The resulting hypothyroidism may lead to hyperlipidaemia even at subclinical stages [38]. Reduction in the levels of GH and thyrotropin during disturbed non-REM sleep may explain our findings. However, this needs to be confirmed by simultaneous measurement of hormones and lipids.

Studies finding differences in REM versus non-REM OSA in the risk for cardiovascular disease, hypertension and diabetes had important clinical implications about the effect of CPAP compliance on comorbidities [14–16, 19]. Theoretically, because REM phase occurs predominantly during the latter stages of sleep, too short (< 4 h) CPAP usage at the beginning of sleep may have markedly lesser beneficial effect on comorbidities. Dorkova et al. compared the effect of 8-week CPAP treatment on lipid profile in patients who daily used their device for more or less than 4 h [39]. Longer CPAP usage had significantly greater effect on total cholesterol, TG, LDL-C and apoB, while no difference was seen on ApoB1 and HDL-C [39]. Although our findings suggest that non-REM sleep has a more pronounced effect on lipid profile, it should still be recommended to use CPAP as long as possible due to two reasons. Firstly, our study did not differentiate between early and late night non-REM sleep, and secondly, REM sleep was also significantly associated with lipid profile, however less strongly than non-REM.

In summary, we found that AHI during REM is as strongly associated with dyslipidaemia as obstructive events in non-REM sleep. Our findings may at least partly explain previous contradictions between studies analysing lipids and apolipoproteins in OSA and may facilitate further research.
